# TCR-Engineered, Customized, Antitumor T Cells for Cancer Immunotherapy: Advantages and Limitations

**DOI:** 10.1100/tsw.2011.10

**Published:** 2011-01-05

**Authors:** Arvind Chhabra

**Affiliations:** Department of Medicine, University of Connecticut Health Center, Farmington, USA

**Keywords:** cancer, immunotherapy, TCR engineering, MHC class I TCR–engineered CD4 T cells

## Abstract

The clinical outcome of the traditional adoptive cancer immunotherapy approaches involving the administration of donor-derived immune effectors, expanded *ex vivo*, has not met expectations. This could be attributed, in part, to the lack of sufficient high-avidity antitumor T-cell precursors in most cancer patients, poor immunogenicity of cancer cells, and the technological limitations to generate a sufficiently large number of tumor antigen-specific T cells. In addition, the host immune regulatory mechanisms and immune homeostasis mechanisms, such as activation-induced cell death (AICD), could further limit the clinical efficacy of the adoptively administered antitumor T cells. Since generation of a sufficiently large number of potent antitumor immune effectors for adoptive administration is critical for the clinical success of this approach, recent advances towards generating customized donor-specific antitumor-effector T cells by engrafting human peripheral blood-derived T cells with a tumor-associated antigen-specific transgenic T-cell receptor (TCR) are quite interesting. This manuscript provides a brief overview of the TCR engineering-based cancer immunotherapy approach, its advantages, and the current limitations.

## INTRODUCTION

Nature has designed our immune system in such a way that it can effectively target invading “foreign” infectious agents, but leave our “self” architecture intact. T cells play a critical role in the generation of an effective antigen-specific immune response. Of the three key components of T-cell immunity — antigen-presenting cells (APC), CD4+ T cells, and CD8+ T cells — APC process and present the antigens to the effector T cells in the form of small peptides bound to the major histocompatibility complex (MHC) molecules, CD8+ T lymphocytes recognize their target epitope in context to the MHC class I molecules, and the CD4+ T lymphocytes recognize their antigenic epitope in context to the MHC class II molecules. Once primed, CD8 T cells acquire antigen-specific lytic function and can recognize and kill the antigen-expressing target cells, such as the tumor cells. CD4 T cells, on the other hand, function as the immune facilitator “helper” cells or the immune-suppressor/regulatory cells.

The success of vaccines against several infectious agents elegantly demonstrated the remarkable specificity, efficacy, and safety of the immune-based therapeutic approaches. As a result, early work in the field of tumor immunology was aimed at developing similar approaches to generate productive antitumor immune responses. Although these studies could not reproduce the success observed in infectious disease settings, a wealth of information was generated that helped us to identify the key factors limiting the success of the current immune-based cancer therapy approaches[1]. We now know that most cancer-associated antigens are "self-antigens". Since most self-reactive high-avidity T cells are deleted during development, it is no more a mystery why a great majority of cancer patients do not harbor sufficient numbers of the high-quality antitumor T-cell precursors, and fail to generate a productive antitumor immune response with the traditional vaccine-based cancer immunotherapy strategies.

We also now know that a growing tumor employs numerous immune-evasion strategies. Among these are the creation of an immunosuppressive environment at the tumor site that results in immune dysfunction of tumor-infiltrating lymphocytes (TIL); down-regulation of the MHC class I molecules on tumor cells, resulting in an inefficient recognition by CD8+ antitumor lytic effectors; and the lack of the MHC class II expression by most human tumors, limiting the simultaneous engagement of CD4+ "helper" response at the tumor site alongside CD8+ "lytic" response. In addition, we now have a better understanding of the CD4+ suppressor/regulatory T cells and their influence on the generation of a productive antitumor immune response[1,2,3,4].

While the active-specific immunization-based approaches were being carefully tested to generate a productive antitumor immune response, approaches were also developed to expand the antitumor immune effectors *ex vivo* and administer them back to the cancer patients, a strategy termed adoptive cancer immunotherapy. Towards this, initial studies employed the lymphokine activated killer (LAK) cells, the nonspecific lytic effectors generated by culturing patient-derived immune effectors in the presence of high-dose interleukin-2[5]. Following the development of the methods to identify, isolate, characterize, and expand tumor-reactive T cells from the tumor site and/or peripheral blood, strategies were developed to administer the antitumor polyclonal populations or tumor antigen–specific clones to the cancer patients[6,7], derived from the peripheral blood–derived antitumor T cells or the TIL of the cancer patients. Although the administration of the tumor antigen–specific clone should inadvertently be a preferred treatment regimen, the generation of such patient-specific T-cell clones is quite a time-consuming and technically demanding process. Furthermore, with most cancer patients lacking sufficient numbers of high-quality antitumor T-cell precursors, these approaches might not produce a broad-scale success[1].

## STRATEGIES TO ENGINEER AN ANTITUMOR IMMUNE RESPONSE

The recent advances in the field of immunology have provided us with a better understanding of the key requirements for the generation of a productive antigen-specific CD8+ cytolytic T-cell response. Among these are the quality of the APC presenting the processed antigen to the effector T cells; the role of a costimulatory molecule–driven "second signal" for an optimum activation of the antigen-specific precursor T cells; and the requirement of cytokines for the survival, maintenance, and proliferation of the antigen-specific T cells. With traditional cancer immunotherapy approaches falling short in producing a productive antitumor immune response, this knowledge was utilized for the development of the strategies that could direct the physiologic non–antitumor immune effectors to function as potent antitumor effectors, and could be administered to the cancer patients regardless of their HLA status, a key requirement of the traditional tumor-associated antigen-specific T-cell–based immunotherapy approaches. These approaches include the development of the bispecific antibodies and the chimeric antigen receptors (CARs).

A bispecific monoclonal antibody mediates the cellular destruction of the tumor cells by binding the tumor target with one domain and the effector cells with the other. Therefore, this approach can simultaneously engage the humoral as well as the cellular arms of the antitumor immunity[8]. The basic principle behind CARs, schematically shown in Fig. 1, is the creation of a hybrid antibody-TCR entity in which the extracellular domain of the CAR is a single-chain antibody-variable fragment (scFv) comprised of the heavy and light variable chains (VH and VL) of a monoclonal antibody, harboring specificity towards a given extracellular antigen (such as CD20, CD19, etc.) that allows the target recognition by the engineered T cells, and in which the intracellular domain of CAR is comprised of the TCR ζ-chain that allows the activation of the T-cell effector function machinery and proliferation following the engagement of the extracellular domain with its target[9,10].

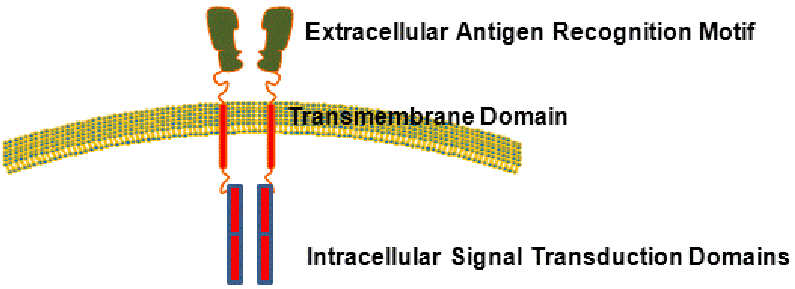

**FIGURE 1.** Schematic representation of the CAR.

To date, more than 40 molecules targeted by CARs have been reported in the literature[10]. Although an interesting concept, the key requirement of CAR-based approaches is the fact that the target must be cell-surface expressed. This could serve as a limitation, as many tumors are known to down-regulate the expression of tumor antigens. In addition, the lack of the costimulatory molecule engagement, a critical requirement for the traditional TCR engagement-driven optimum T-cell activation, could further limit the efficacy of the CAR-engineered immune effectors, by making them anergic. Furthermore, in the absence of costimulatory signals, the signal triggered by the engagement of CARs might not be as optimum a signal as relayed by the natural TCR. In this context, the development of the next-generation CARs that also contain costimulatory immune components is quite significant[11,12]. These modified CARs could very well address the concerns associated with the first-generation CARs.

Many clinical trials have been carried out[10,13,14] and many more are under way to systematically examine the safety and clinical efficacy of the CAR-engineered lymphocytes. These studies would provide key insights on the clinical potential of CAR-based approaches. In addition, efforts are also under way to generate customized antitumor immune responses for adoptive cancer immunotherapy by TCR engineering.

## TCR ENGINEERING–BASED APPROACHES FOR CANCER IMMUNOTHERAPY

Since the functional specificity of the antigen-specific T cells is determined by the TCR they express, with the development of the technical know-how to isolate TCR and reprogram the TCR-engrafted T cells in the mouse model[15], several laboratories aimed to isolate the TCRs from the tumor antigen–specific T cells in order to create customized antitumor T cells[16,17,18,19]. The basic principle behind this strategy, shown schematically in Fig. 2, is that with the availability of a transgenic TCR of the desired tumor-antigenic specificity and functional avidity, effector T cells derived from the cancer patients of an identical HLA subtype could be engrafted and programmed to function as customized antitumor immune effectors, and by *in vitro* expansion methods, sufficient tumor-reactive T cells could be generated for adoptive administration to the cancer patients.

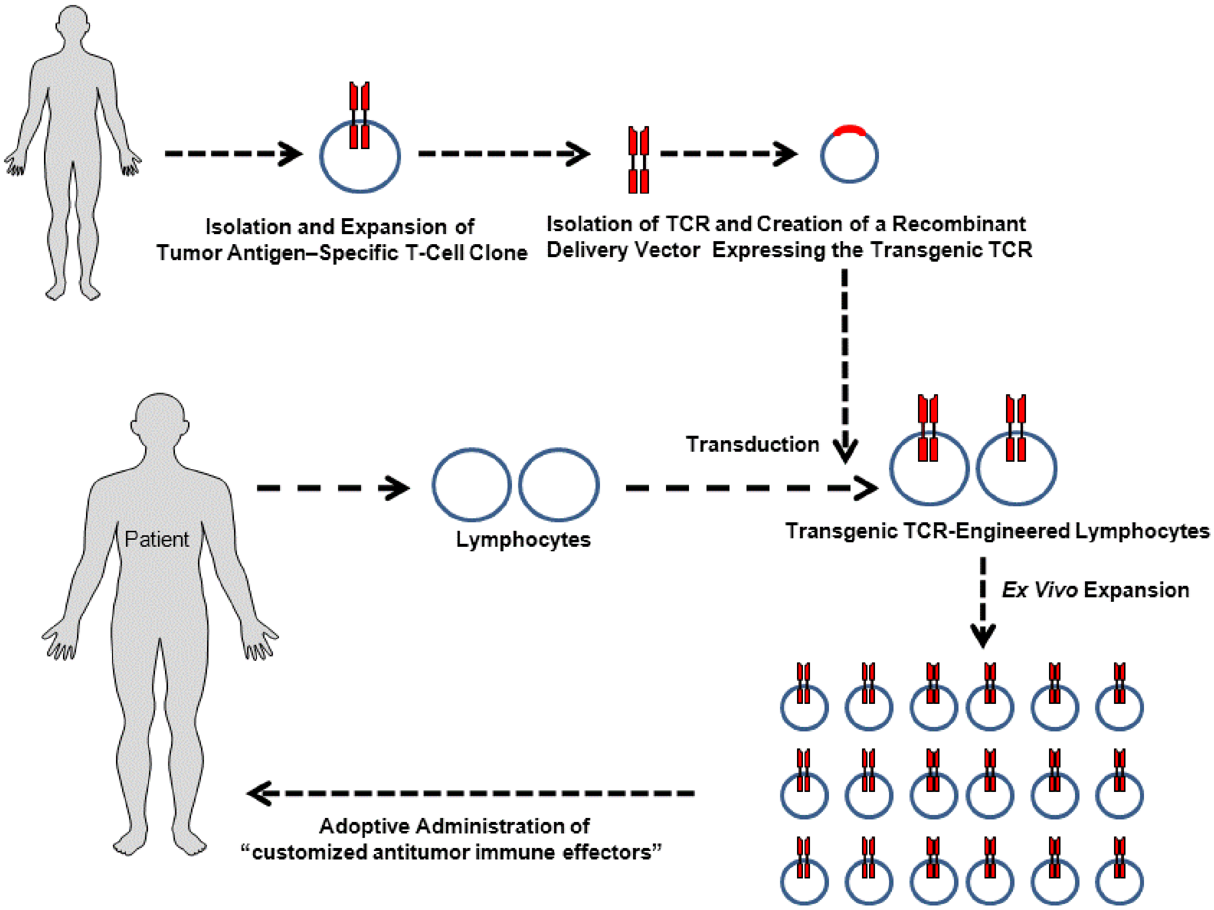

**FIGURE 2.** Schematic representation of the TCR engineering–based adoptive cancer immunotherapy approach.

These efforts led to the isolation of the first human tumor-associated antigenic epitope–specific TCR in a human melanoma model in 1994[16]. Since then, several TCRs with diverse functional specificities have been isolated, and methods have been generated to effectively engineer T cells with the transgenic TCRs and customized T cells that can exhibit potent antitumor responses[17,18,19,20,21,22]. This approach has also been successfully utilized to create immune effectors that harbor dual functional specificities against viral and tumor antigens[17]. Although the TCR engineering approach is conceptually quite straightforward, a careful comparative analysis of the different studies reporting the isolation of tumor antigen–specific TCRs and the functional characterization of the TCR-engineered T cells reveals that different transgenic TCRs produce quite different functional profiles. In many of these studies, whole PBL were transduced with the transgenic TCR and they were shown to exhibit mixed Th1/Th2 effector cytokine profiles with varying degrees of antigen-specific cytolytic function[20,21]. Therefore, it is critical to identify a transgenic TCR of ideal affinity and functional avidity that can produce the desired functional outcome.

Evidently, while the tumor immunology field was making steady progress in the last decade or two, studies in infectious disease models established a critical requirement of CD4 T cells in the generation of a productive CD8+ CTL response[23,24]. As a result, we now know that for the development of a successful T-cell–based cancer immunotherapy, the engagement of the CD4+ "helper" arm of T-cell immune response is essential[19,25]. Although the main rationale behind the development of the TCR engineering–based approaches was to create CD8+-customized lytic effectors, as discussed below, recent data suggest that this approach could also be utilized to engage the CD4 T cells in tumor immunity, in an antigen-specific manner, a task quite challenging in normal physiology given the fact that most human tumors do not express MHC class II molecules.

## ROLE OF CD4 T CELLS IN TUMOR IMMUNITY AND APPLICATION OF THE TCR ENGINEERING APPROACH TO ENGAGE CD4 T CELLS IN CANCER IMMUNOTHERAPY PROTOCOLS

As mentioned above, CD8+ T cells have long been considered as the key determinants of an effective T-cell response against infectious agents as well as cancer. Therefore, a great majority of effort in the tumor immunology field has been directed towards the identification and characterization of the MHC class I–restricted epitopes of tumor-associated antigens, and the development of strategies to generate a productive CD8+ antitumor cytolytic T-lymphocyte (CTL) response. However, several laboratories have shown that CD4 T cells also play a critical role in tumor immunity[25,26,27,28]. Studies in mouse models have shown that CD4 T cells can not only contribute in tumor immunity as the “helper effectors” to facilitate the generation of a productive CD8+ CTL response, but also as the direct antitumor effectors[25,26,27,29]. In clinical settings also, CD4 T cells engineered with tumor-associated antigenic epitope–specific TCR have been shown to produce an impressive antitumor response in cancer patients[30]. More recently, utilizing the gp75/tyrosinase-related protein (TRP)-1–specific CD4+ transgenic mouse model that harbors class II–restricted T cells, two studies provide additional evidence that the CD4 T cells can function as MHC class II–restricted lytic effectors and can produce an impressive antitumor response[31,32], which could be further boosted by incorporating an anti–CTLA-4 blockade approach[32]. It should be emphasized here that the TCRs utilized in these studies were MHC class II restricted, the natural physiological TCRs associated with the CD4 T cells[30,31,32].

These studies highlighted the urgent need to develop methodologies to engage CD4 T cells in tumor immunity. However, for most tumor antigens, and for any given HLA subtype, MHC class II–restricted epitopes have not yet been characterized. Therefore, it is conceptually quite difficult to simultaneously engage CD4 and CD8 T cells in cancer immunotherapy for a majority of tumor antigens. In this context, recent data from our group showing that a MHC class I–restricted TCR can effectively program human CD4 T cells to function as MHC class I–directed antitumor effectors that would not only exhibit a MHC class I–directed “helper response”, but also produce an antigen-specific “lytic function” of their own, are quite interesting[33]. In a follow-up study, we found that these engineered T cells are multifunctional[34], a property associated with the superior antitumor response in different antigenic models[35,36]. This approach makes it feasible to simultaneously engage the CD4 and the CD8 T cells in antitumor T-cell immunity, in an antigen-specific manner[19]. However, it should be mentioned here that different transgenic TCRs reported in the literature have been shown to not only vary in the surface expression profiles in the engineered cells, but the functional profiles of the TCR-engineered T cells are also quite variable[16,17,18,19]. Taking our findings in context with the effector functional profiles described for engineered T cells with different transgenic TCRs[20,21], it should be emphasized here that in order to successfully apply this approach to different tumor models, identification and characterization of a MHC class I–restricted transgenic TCR that would not only program the engrafted CD8 T cells to function as customized lytic effectors, but also would allow CD4 T cells to function as MHC class I–directed “helper as well as lytic effectors”, is essential.

## TCR-ENGINEERED T CELLS: CURRENT LIMITATIONS

The methodology to generate sufficient numbers of patient-specific TCR-engineered customized effector T cells is conceptually a pretty straightforward process. However, several factors need to be addressed to make this approach a clinical success. Systematic studies are needed to fully understand the biology of the transgenic TCR-engineered CD4+ and CD8+ effector T cells. Methodologies need to be perfected in order to increase paring of transgenic TCR chains; for example, by introducing additional cystein bonds, by utilizing the mouse TCR constant region, and by introducing leucine zipper sequences in the transgenic TCR chains. Additional studies are also needed in order to fully understand the role of endogenous TCR on the pairing of the transgenic TCR chains, the possibility of TCR mixing, and the probability of the creation of novel autoreactive TCRs (Fig. 3). Such studies are crucial because despite the preliminary encouraging results in cancer patients showing that the TCR-engineered immune effectors are well tolerated[37], a recent study in a mouse model of TCR gene therapy suggests the development of a potentially lethal graft-vs.-host disease[38].

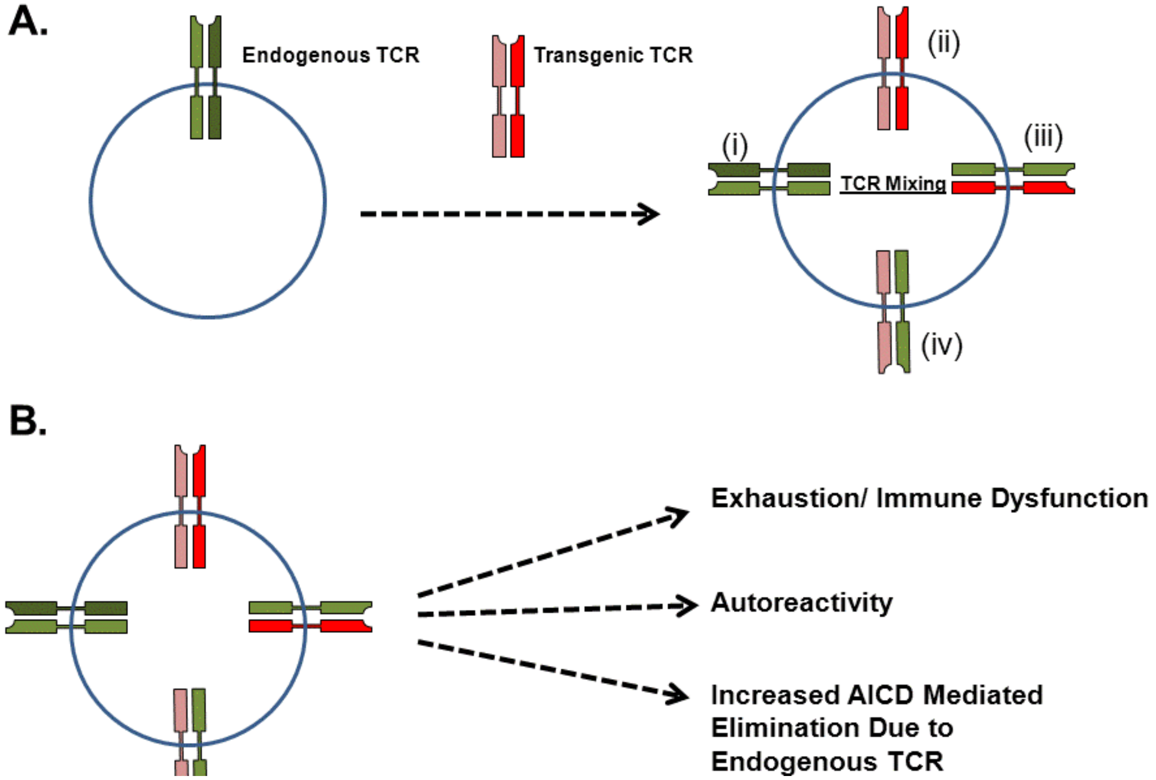

**FIGURE 3.** Cartoon showing the limitations of the TCR engineering approach. (A) Possible novel hybrid TCR sets created by mixing of the α- and β-chains of the endogenous TCR and the transgenic TCR. Such mixed TCR-expressing immune cells could exhibit a potentially harmful autoreactivity, depicted in Fig. 3B. (B) TCR-engineered cells are also susceptible to the historic barriers of cancer immunotherapy; for example, the tumor microenvironment–mediated immune dysfunction and the epitope-specific AICD-mediated premature elimination.

Strategies are also needed that can better prepare the TCR-engineered T cells to overcome the historical factors limiting the success of cancer immunotherapy approaches, such as the tumor microenvironment–mediated immune dysfunction and/or epitope-specific AICD-mediated elimination of a significant fraction of the adoptively administered antitumor effectors. Therefore, concerted efforts are needed in order to develop the next-generation TCR-engineered antitumor immune effectors that would be less susceptible to undergo immune dysfunction at the tumor site, due to the immunosuppressive tumor microenvironment. It is also feasible to develop antitumor immune effectors that would be capable of not only withstanding the host regulatory mechanisms, such as nReg-mediated immune suppression and/or conversion to iReg phenotypes in immunosuppressive tumor microenvironment; but also would better withstand the immune homeostasis mechanisms, such as the epitope-specific AICD-mediated elimination[39]. In this context, the combinatorial approaches, such as the incorporation of CTLA-4 and/or PD-1 blockade, etc. that have produced promising results in preclinical studies[32], have sufficient merit to be carefully tested in clinical settings.

Last but not least, despite the development of next-generation retro- and lentiviral vectors, the use of viral vectors has long been a therapeutic concern. Therefore, it will be interesting to develop novel nonviral methods that will offer a superior transduction efficiency and lack of viral gene introduction into cancer patients. The introduction of the suicide genes in TCR-engineered T cells is also an interesting approach, as this would allow us to get rid of the adoptively administered immune effectors, if needed, for example, in the case of an adverse event.

## CONCLUSIONS

The recent FDA approval of the first cancer vaccine for prostate cancer has generated a lot of excitement in the cancer immunotherapy field[40]. The encouraging results observed in a limited number of cancer patients with the current adoptive immunotherapy approaches, such as with the TCR-engineered T cells, have further added to this enthusiasm. These developments offer a renewed hope that the development of a reliable patient-specific immune-based cancer therapy would soon become a reality, and not just remain a distant goal. However, novel nonviral methodologies are urgently needed in order to engineer the host immune effectors reliably and reproducibly. Systematic studies are also required to fully comprehend the biology, safety, and clinical efficacy of the transgenic TCR-engineered CD4 and CD8 T cells. In addition, the development of the next-generation engineered T cells, through TCR engineering–based approaches or through modified CARs, which could overcome/withstand the historical limiting factors in the field of cancer immunotherapy, such as immune dysfunction, would also be critical in order to develop a T-cell–based cancer immunotherapy approach as a reliable treatment modality for the masses.

## ACKNOWLEDGMENTS

The author thanks all the contributors of the studies that have been cited here and many more whose elegant works have contributed immensely towards advancing the field, but could not be cited here only because of the space limitations. Grant support from the Breast Cancer Alliance, Greenwich, Connecticut, and the State of Connecticut Stem Cell Research Initiative are also duly acknowledged.
